# Identification of hospitalized elderly patients at risk for adverse in-hospital outcomes in a university orthopedics and trauma surgery environment

**DOI:** 10.1371/journal.pone.0187801

**Published:** 2017-11-10

**Authors:** Janine Gronewold, Christian Dahlmann, Marcus Jäger, Dirk M. Hermann

**Affiliations:** 1 Department of Neurology, University Hospital Essen, University of Duisburg-Essen, Essen, Germany; 2 Department of Nursing Research, University Hospital Essen, University of Duisburg-Essen, Essen, Germany; 3 Department of Orthopedics and Trauma Surgery, University Hospital Essen, University of Duisburg-Essen, Essen, Germany; National Yang-Ming University, TAIWAN

## Abstract

**Background:**

As a consequence of demographic changes, hospitals are confronted with increasing numbers of elderly patients, who are at high risk of adverse events during hospitalization. Geriatric risk screening followed by comprehensive geriatric assessment (CGA) and treatment has been requested by geriatric societies and task forces to identify patients at risk. Since empirical evidence on factors predisposing to adverse hospital events is scarce, we now prospectively evaluated implications of geriatric risk screening followed by CGA in a university hospital department of orthopedics and trauma surgery.

**Methods:**

Three hundred and eighty-one patients ≥75 years admitted to the Department of Orthopedics and Trauma Surgery of the University Hospital Essen received Identification of Seniors at Risk (ISAR) Screening followed by CGA via a geriatric liaison service in case of positive screening results. Associations between ISAR, CGA, comorbid risk factors and diseases, length of hospital stay, number of nursing and physiotherapy hours, and falls during hospital stay were analyzed.

**Results:**

Of 381 ISAR screenings, 327 (85.8%) were positive, confirming a high percentage of patients at risk of adverse events. Of these, 300 CGAs revealed 82.7% abnormal results, indicating activities of daily living impairment combined with cognitive, emotional or mobility disturbances. Abnormal CGA resulted in a longer hospital stay (14.0±10.3 days in ISAR+/CGA abnormal compared with 7.6±7.0 days in ISAR+/CGA normal and 8.1±5.4 days in ISAR-, both p<0.001), increased nursing hours (3.4±1.1 hours/day in ISAR+/CGA abnormal compared with 2.5±1.0 hours/day in ISAR+/CGA normal and 2.2±0.8 hours/day in ISAR-, both p<0.001), and increased falls (7.3% in ISAR+/CGA abnormal, 0% in ISAR+/CGA normal, 1.9% in ISAR-). Physiotherapy hours were only significantly increased in ISAR+/CGA abnormal (3.0±2.7 hours) compared with in ISAR+/CGA normal (1.6±1.4 hours, p<0.001) whereas the comparison with ISAR- (2.4±2.4 hours) did not reach significance (p = 0.368). In multivariable regressions, the CGA domains activities of daily living impairment (assessed by Barthel-Index) and signs of depression (assessed by geriatric depression scale) predicted longer length of hospital stay. High ISAR score, and impairment in activities of daily living and cognition (assessed by mini-mental state examination and clock-drawing test) predicted increased nursing hours, and impairment in activities of daily living and mobility predicted increased physiotherapy hours.

**Conclusions:**

An abnormal geriatric screening and assessment is associated with longer hospital stay, more nursing and physiotherapy hours, and more falls.

## Introduction

As a consequence of demographic changes which are related to decreasing birth rates and increasing life expectancy, hospitals are confronted with an increasing number of elderly patients. When elderly people are admitted to hospital because of an acute health event, they are at high risk of adverse outcomes during and after hospitalization[1]. This implies a decline in the ability to perform activities of daily living because of reduced physical, cognitive or emotional functioning. During acute hospital admission, routine care mainly concentrates on diagnostic and therapeutic interventions targeting the acute illness while comorbid geriatric problems leading to functional decline are often overlooked. Functional decline represents a huge social and economic burden. Outcomes of elderly patients can be improved by comprehensive geriatric assessment (CGA) and intervention[2,3]. For optimal resource management, careful selection of patients who will benefit from this approach is important. Several instruments were developed to identify such people at risk of functional decline of which the Identification of Seniors at Risk questionnaire (ISAR)[4] is most frequently used because it has been validated in different cohorts[5]. ISAR is short and easy to administer and does not require specialized training. Acceptable validity and reliability for ISAR has already been reported in emergency departments[6,7], but it was never assessed in other hospital environments in combination with CGA.

In view of further population aging, systematic risk screening and assessment of age-related cognitive, emotional and mobility impairments has been demanded to be imperative by various national geriatric societies such as the American Geriatric Emergency Medicine Task Force[8], the Australian and New Zealand Society for Geriatric Medicine[9], and collaborations such as the recent European Taskforce on Geriatric Emergency Medicine[10]. However, those suggestions are still based on rather limited empirical evidence and so far studies mostly concentrated on the emergency department setting[6,7]. In order to close this gap, we now evaluated the prevalence of a positive ISAR screening and abnormal CGA results and their association with the duration of hospital stay, nursing and physiotherapy hours, and falls in a university hospital department of orthopedics and trauma surgery. This department receives a high number of elderly patients admitted due to injuries. Beside cardiovascular disorders, degenerative diseases such as osteoarthritis are associated with falls in the elderly. Previous studies showed that about 60% of all emergencies in elderly people result from injuries due to falls[11], rendering a department of orthopedics and trauma surgery an ideal environment for the evaluation of age-related patient risks.

## Materials and methods

### Study design

This is a prospective cross-sectional single-center cohort study. Three hundred and eighty-one patients aged ≥75 years (inclusion criterion) consecutively admitted to the Department of Orthopedics and Trauma Surgery of the University Hospital Essen between July 2015 and April 2016 received geriatric risk screening and CGA. The aim was to evaluate the prevalence of abnormal geriatric risk screening and CGA and their consequences for the duration of hospital stay, nursing and physiotherapy hours and falls. The study was approved by the ethical committee of the University Duisburg-Essen and need for consent was waived. We chose the Department of Orthopedics and Trauma Surgery because of the high percentage of hospitalized elderly patients (24.8% ≥75 years compared with 15.0% in the whole University Hospital Essen during the study period). In accordance with a previous German validation study we did not exclude patients living in long-term care facilities[6]. Clinical information and information on medical histories were collected from patient records.

### Identification of Seniors at Risk (ISAR) screening

ISAR was developed in community-dwelling patients aged ≥65 years admitted to the emergency departments of four acute-care hospitals in Montreal, Canada[4] and later adapted[12] to predict a composite measure of adverse health outcome during 6 months of follow-up including death, admission to a nursing home or long-term hospitalization, or a clinically significant decrease in functional status. In the original version, ISAR consists of 6 questions with dichotomized yes/no answers assessing functional dependence (premorbid and acute change), recent hospitalization, impaired memory, impaired vision and polypharmacy. Different cut-offs have been suggested, but a score ≥2 is regarded to result in the best balance between sensitivity and specificity for the prediction of adverse health outcomes[7]. In our study, the adapted ISAR version was used[12]. Nurses were trained in performing the ISAR screening by the geriatric liaison service team consisting of a geriatrician, an occupational therapist and a psychologist. Screening forms were integrated into the electronical Hospital Information System (HIS) Cerner medico and were filled in on occasion of the admission interview based on the patients’ personal information and available medical reports. In line with previous suggestions[7], an ISAR score ≥2 was regarded positive (ISAR+) and triggered a CGA while scores <2 were regarded as normal (ISAR-) and had no consequences. ISAR screening took place on all days of the week 24 hours per day.

### Comprehensive geriatric assessment (CGA)

Patients with positive ISAR screenings received a CGA by the geriatric liaison service team as soon as possible after ISAR screening. CGAs were performed from Monday to Friday during 9 am to 5 pm using a standardized geriatric test battery assessing impairment in activities of daily living (Barthel-Index[13]), mobility (timed Up & Go[14], Tinetti mobility test[15]), cognition (mini-mental state examination test (MMSE)[16], clock-drawing test[17]) and signs of depression (15-item short form of the geriatric depression scale (GDS)[18]). The Barthel-Index uses 10 items to measure dependence in activities of daily living including help needed with eating, transfer (e.g. from bed to chair), grooming, bathing, toileting, walking, climbing stairs and dressing as well as presence of anal and urinary incontinence. The resulting score ranges from 0–100, in line with published suggestions on how to perform and evaluate geriatric assessments[19] we defined a score <90 as impairment in activities of daily living. The timed Up & Go test measures the time a person needs to rise from a chair, walk three meters, turn around, walk back to the chair, and sit down again. The test should be completed in <20 seconds because this is the limit to cross the road during the green phase of the traffic light, longer times needed indicate that the person needs assistance outside and are defined as mobility impairment as well as when the patient was not able to perform the test for example due to fractures or bed rest after emergency operation. The Tinetti mobility test is composed of two sections assessing a person's static balance abilities while sitting and standing as well as dynamic balance abilities while walking. Scores <20 are defined as mobility impairment and indicate gait and postural disturbance, mobility impairment is also scored when the patient was not able to perform the test. The MMSE is widely used as a dementia screening and assesses orientation to time and place, memory, calculation, language functions (naming, repetition, following complex instructions, reading, writing), motor skills and visuoconstruction. A score of <28 is defined as impairment in cognition ranging from mild cognitive impairment to severe dementia. The clock-drawing test is also used as a short dementia screening, which asks patients to draw a clock showing the time 11.10 am. According to the criteria by Shulman et al. 1993[17], a score ≥3 is regarded as indicative of impairment in cognition. The 15-item version of the GDS by Yesavage[18] is extensively used as a screening tool for depression in the clinical setting in the elderly population. A score ≥6 suggests signs of depression. Abnormal CGA is defined as significant impairment in activities of daily living (Barthel-Index <90) combined with deficits in the CGA domains mobility (timed Up & Go ≥20 seconds or Tinetti mobility test score <20), cognition (MMSE test score <28 or clock-drawing test score ≥3) or signs of depression. Based on CGA results, patient management suggestions to responsible physicians were made.

### Nursing and physiotherapy workload

Nursing and physiotherapy workload was quantified using routine data from the HIS. Operationalization of nursing workload at the University Hospital Essen is performed using the “LEP” (“Leistungserfassung in der Pflege”) catalogue, a set of approximately 180 variables covering all aspects of nursing inpatient care. Each variable includes a time value, which is coded as the default value or adapted by the nurse who conducted the measure. Operationalization of physiotherapy workload was performed using data from the HIS electronic treatment documentation system.

### Falls

Data about accidental in-hospital falls were also retrieved from the HIS electronic documentation system. All accidental falls at the University Hospital Essen are documented using an electronic form, which does not only provide the structure of information to be documented but is also used for triggering orders to nursing experts who evaluate each fall.

### Statistical analysis

Continuous data are presented as mean±SD values, categorical data as counts (%). Comparisons between ISAR-, ISAR+/CGA normal and ISAR+/CGA abnormal groups were done with one-way ANOVA followed by Games Howell post-hoc tests for continuous and with Chi-square or Fisher’s exact tests for categorical data. To evaluate predictors of different outcomes (length of hospital stay, hours of nursing and physiotherapy as well as number of falls), uni- and multivariable linear regressions (forced entry method) were calculated. The factors age, sex, number of medical diagnoses at admission, ISAR score, activities of daily living, mobility and cognition impairment as well as signs of depression were first inserted unadjusted into these regressions. In a next step we analyzed the influence of (a) age, sex, and ISAR score (model 1), (b) age, sex, mobility and cognition impairment and signs of depression (model 2), (c) age, sex, activities of daily living and cognition impairment and signs of depression (model 3), (d) age, sex, activities of daily living, mobility and cognition impairment and signs of depression (model 4), (e) age, sex, ISAR score, activities of daily living, mobility and cognition impairment and signs of depression (model 5) and (f) age, sex, number of medical diagnoses at admission, ISAR score, activities of daily living, mobility and cognition impairment and signs of depression (model 6) on the different patient outcomes. P values <0.05 indicate statistical significance and are shown in bold in the tables. All statistics were performed using SPSS19 for Windows (SPSS, Chicago, IL, U.S.A.).

## Results

### Study cohort

Within the study period between July 2015 and April 2016, 1703 patients were admitted to the Department of Orthopedics and Trauma Surgery. Of those, 423 (24.8%) were ≥75 years. 381 patients (90.1%) received an ISAR screening (95 men, 286 women, mean age 82.5±5.5 years, range 75–102 years). Of all ISAR screenings, 327 (85.8%) were positive (ISAR ≥2). Of 327 ISAR+ patients, 300 (91.7%) received CGA. 27 patients could not be examined due to reasons of transfer, discharge, foreign-language or non-compliance. Missing data due to reasons of transfer or discharge occurred when patients arrived at the hospital Friday evening or at the weekend outside the working hours of the geriatric liaison service team and were rapidly transferred to the operation room in case of urgent conditions or discharged home in case of minor illness conditions like mild concussion. Missing data due to foreign-language patients occurred when no relative was present for translation into the mother language of the patients because CGAs by the geriatric liaison service team could only be performed in German or English language. In very rare cases, patients refused to participate in CGA.

Of 300 CGAs performed, 248 patients (82.7%) revealed significant impairment in activities of daily living (Barthel-Index <90) combined with deficits in the CGA domains mobility (timed Up & Go ≥20 seconds or Tinetti mobility test score <20), cognition (MMSE test score <28 or clock-drawing test score ≥3) or signs of depression (GDS ≥6; see section ‘comprehensive geriatric assessment’) (in the following referred to as abnormal CGA result). Patients with ISAR+/CGA abnormal were significantly older than those with ISAR+/ CGA normal (p = 0.003) and those with ISAR- (p<0.001) with no significant difference between ISAR+/ CGA normal and ISAR- (Table 1). In general, ~75% of patients ≥75 years admitted to the Department of Orthopedics and Trauma Surgery were female with a higher percentage in the ISAR+/CGA abnormal than in the ISAR- group (p = 0.036). In ISAR+/CGA abnormal patients, the prevalence of cardiac diseases, namely atrial fibrillation, coronary heart disease, heart failure and history of myocardial infarction as well as the percentage of known diabetes and dementia was significantly higher compared with ISAR- patients (p = 0.013, 0.007, 0.006, 0.050 and 0.044, respectively).

**Table 1:** Characteristics of the total study cohort also split by ISAR score and CGA results.

	Total (n = 381)	ISAR- (n = 54; 15.2%)	ISAR+/CGA normal (n = 52; 14.7%)	ISAR+/CGA abnormal (n = 248; 70.1%)	p-value ISAR+/CGA normal vs ISAR-	p-value ISAR+/CGA abnormal vs ISAR-	p-value ISAR+/CGA abnormal vs ISAR+/CGA normal
Age (years)	82.5±5.5	79.3±3.8	80.8±4.7	83.4±5.5	0.185	**<0.001**	**0.003**
Sex (male)	95(24.9)	20(37.0)	12(23.1)	57(23.0)	0.138	**0.036**	0.999
Anemia	18(4.7)	0	0	18(7.3)	0.999	0.051	0.051
Chronic kidney disease	55(14.4)	6(11.1)	5(9.6)	43(17.3)	0.999	0.315	0.213
Heart failure	45(11.8)	1(1.9)	4(7.7)	39(15.7)	0.205	**0.006**	0.190
Coronary heart disease	64(16.8)	3(5.6)	10(19.2)	48(19.4)	**0.015**	**0.007**	0.999
Atrial fibrillation	70(18.1)	4(7.4)	8(15.4)	56(22.6)	0.236	**0.013**	0.272
Other cardiac arrhythmias	32(8.4)	2(3.7)	4(7.7)	26(10.5)	0.437	0.190	0.623
Valve insufficiency	34(8.9)	3(5.6)	3(5.8)	26(10.5)	0.999	0.322	0.325
Chronic obstructive pulmonary disease	24(6.3)	0	4(7.7)	17(6.9)	0.057	0.092	0.999
Peripheral artery disease	11(2.9)	2(3.7)	1(1.9)	6(2.4)	0.999	0.634	0.999
Arterial hypertension	260(68.2)	40(74.1)	34(65.4)	169(68.1)	0.402	0.514	0.745
Diabetes	79(20.7)	6(11.1)	9(17.3)	60(24.2)	0.416	**0.044**	0.365
Hyperlipoproteinemia	23(6.0)	3(5.6)	2(3.8)	17(6.9)	0.999	0.782	0.545
Nicotine abuse	5(1.3)	0	1(1.9)	4(1.6)	0.495	0.603	0.999
Obesity	12(3.1)	0	2(3.8)	8(3.2)	0.243	0.359	0.686
History of myocardial infarction	25(6.6)	1(1.9)	6(11.5)	17(6.9)	**0.013**	**0.050**	0.254
History of pulmonary embolism	10(2.6)	0	3(5.8)	7(2.8)	0.118	0.361	0.386
History of stroke	27(7.1)	2(3.7)	3(5.8)	21(8.5)	0.678	0.279	0.778
History of thrombosis	14(3.7)	2(3.7)	3(5.8)	9(3.6)	0.678	0.999	0.443
Hyperthyroidism	5(1.3)	2(3.7)	1(1.9)	2(0.8)	0.999	0.144	0.436
Hypothyroidism	45(11.8)	5(9.3)	5(9.6)	32(12.9)	0.999	0.510	0.646
Dementia	69(18.1)	1(1.9)	5(9.6)	53(21.4)	0.113	**0.001**	0.054
Alcohol abuse	12(2.1)	0	0	10(4.0)	0.999	0.219	0.220
Depression	23(6.0)	3(5.6)	2(3.8)	12(4.8)	0.999	0.999	0.999
Anxiety disorder	5(1.3)	0	1(1.9)	4(1.6)	0.495	0.603	0.999
Parkinson’s disease	14(3.7)	3(5.6)	1(1.9)	9(3.6)	0.618	0.449	0.999
Polyneuropathy	13(3.4)	3(5.6)	2(3.8)	6(2.4)	0.999	0.369	0.631
Cancer	53(13.9)	8(14.8)	4(7.7)	38(15.3)	0.359	0.999	0.189
Cataract	15(3.9)	1(1.9)	2(3.8)	12(4.8)	0.618	0.477	0.999
Presbyacusia	8(2.1)	2(3.7)	0	6(2.4)	0.495	0.634	0.595
Anal incontinence	2(0.5)	0	0	1(0.4)	0.999	0.999	0.999
Urinary incontinence	11(2.9)	1(1.9)	0	7(2.8)	0.999	0.999	0.609
Decubitus	7(1.8)	0	1(1.9)	6(2.4)	0.495	0.379	0.999

CGA, comprehensive geriatric assessment; ISAR, Identification of Seniors at Risk. ISAR+, positive ISAR screening (score ≥2); ISAR-, negative ISAR screening (score <2). In 327 ISAR+ patients, 300 CGAs were performed (27 missing due to transfer, discharge, foreign-language or incompliance of patients). For definition of abnormal CGA see section ‘Comprehensive geriatric assessment’ in ‘Materials and Methods‘.

Among medical diagnoses, femoral neck fracture due to falls was the most common cause of hospital admission (10.8%), followed by femoral shaft fracture (9.7%), spinal fracture (8.7%), humerus fracture (7.1%), radius fracture (6.6%) and spinal pain (6.0%, Table 2). As expected, femoral neck fracture and femoral shaft fracture were more often associated with abnormal CGA results compared to the other diagnoses. In accordance with the high percentage of fractures leading to hospital admission, 59.6% of the patients were admitted as emergency.

**Table 2:** Medical main diagnosis of the total study cohort also split by ISAR score and CGA results.

Diagnosis	Total (n = 381)	ISAR- (n = 54; 15.2%)	ISAR+/CGA normal (n = 52; 14.7%)	ISAR+/CGA abnormal (n = 248; 70.1%)
Acetabulum fracture	4(1.0)	0	0	4(1.6)
Femoral neck fracture	41(10.8)	2(3.7)	0	38(15.3)
Femoral shaft facture	37(9.7)	4(7.4)	0	29(11.7)
Patella fracture	2(0.5)	2(3.7)	0	0
Tibial or fibular fracture	13(3.4)	0	0	10(4.0)
Ankle fracture	9(2.4)	3(5.6)	0	6(2.4)
Tarsal bone fracture	2(0.5)	0	0	2(0.8)
Metatarsal fracture	1(0.3)	0	0	1(0.4)
Clavicular fracture	1(0.3)	0	0	0
Humerus fracture	27(7.1)	2(3.7)	8(15.4)	15(6.0)
Radius fracture	25(6.6)	3(5.6)	4(7.7)	17(6.9)
Ulnar fracture	14(3.7)	3(5.6)	5(9.6)	5(2.0)
Metacarpal fracture	1(0.3)	0	0	0
Finger fracture	2(0.5)	1(1.9)	0	1(0.4)
Spinal fracture	33(8.7)	5(9.3)	2(3.8)	22(8.9)
Rib fracture	5(1.3)	1(1.9)	1(1.9)	3(1.2)
Pelvic fracture	1(0.3)	0	0	1(0.4)
Basal skull fracture	4(1.0)	1(1.9)	0	2(0.8)
Pathological fracture	6(1.6)	1(1.9)	1(1.9)	4(1.6)
Bone tumor	10(2.6)	2(3.7)	0	8(3.2)
Joint dislocation	3(0.8)	0	1(1.9)	1(0.4)
Mechanical complication or loosening of joint replacement	5(1.3)	1(1.9)	0	4(1.6)
Mechanical complication osteosynthesis	7(1.8)	1(1.9)	1(1.9)	5(2.0)
Infection joint replacement	8(2.1)	0	1(1.9)	7(2.8)
Septic arthritis	1(0.3)	1(1.9)	0	0
Articular effusion	2(0.5)	0	0	2(0.8)
Meniscus damage	1(0.3)	1(1.9)	0	0
Open wound	4(1.0)	0	0	4(1.6)
Ulcus cruris	2(0.5)	1(1.9)	0	1(0.4)
Postoperative hematoma	1(0.3)	0	0	1(0.4)
Bursitis	1(0.3)	0	0	1(0.4)
Discitis	6(1.6)	0	1(1.9)	5(2.0)
Erysipela	2(0.5)	0	0	2(0.8)
Abscess	1(0.3)	0	0	1(0.4)
Pseudarthrosis	4(1.0)	1(1.9)	0	3(1.2)
Osteomyelitis	2(0.5)	1(1.9)	0	1(0.4)
Phlegmone	1(0.3)	0	0	1(0.4)
Omarthrosis	1(0.3)	0	0	1(0.4)
Gonarthrosis	2(0.5)	0	1(1.9)	1(0.4)
Coxarthrosis	5(1.3)	2(3.7)	0	3(1.2)
Spinal stenosis	11(2.9)	4(7.4)	2(3.8)	4(1.6)
Spinal pain	23(6.0)	5(9.3)	11(21.2)	7(2.8)
Intervertebral disc displacement	4(1.0)	1(1.9)	2(3.8)	1(0.4)
Cerebral concussion	20(5.2)	3(5.6)	9(17.3)	6(2.4)
Subdural hematoma	2(0.5)	1(1.9)	0	1(0.4)
Subarachnoid hemorrhage	3(0.8)	0	0	2(0.8)
Early trauma complication	3(0.8)	1(1.9)	1(1.9)	1(0.4)
Hemopneumothorax	1(0.3)	0	0	1(0.4)
Bruise	16(4.2)	0	1(1.9)	12(4.8)
Muscle disorders	1(0.3)	0	0	1(0.4)

Abbreviations as in Table 1.

**Fig 1:**
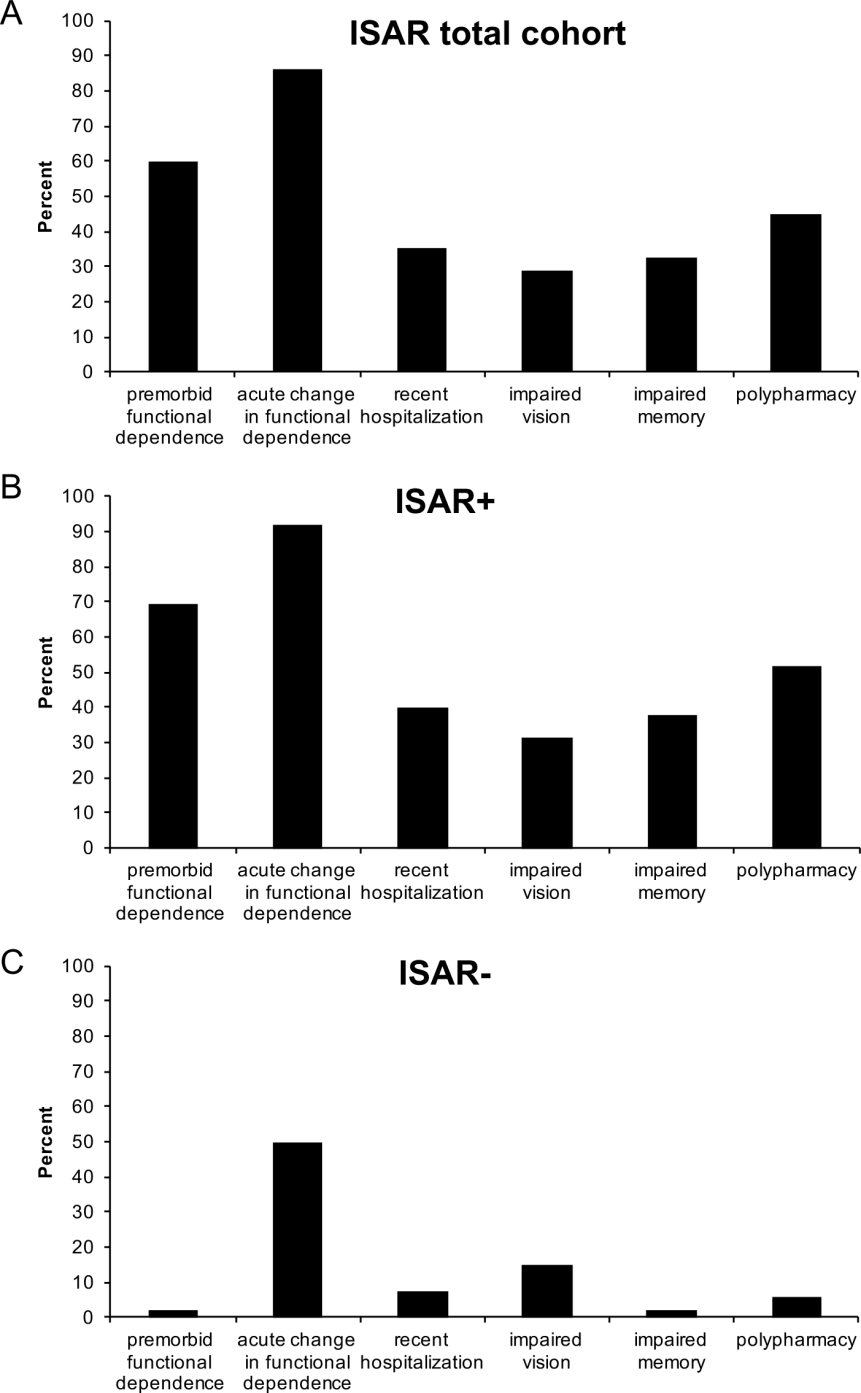
**Prevalence of ISAR items (A) for the total cohort, (B) for ISAR+ and (C) for ISAR-.** Abbreviations as in Table 1.

In the ISAR screening, premorbid functional dependence (59.8%), acute change in functional dependence (86.1%) and polypharmacy (45.1%) were the items that have been most frequently reported, while recent hospitalization (35.2%), impaired vision (28.9%) and impaired memory (32.5%) were less prevalent (Fig 1A). In ISAR+ patients, the pattern was similar to the whole cohort (Fig 1B), while in ISAR- patients acute change in functional dependence was most frequently recorded with all other items revealing prevalence rates below 15% (Fig 1C).

In the CGA, 85% of patients had activities in daily living impairment (Barthel-Index <90), 84% mobility impairment (Timed Up & Go ≥20 seconds in 76%, Tinetti Mobility Test score <20 in 81%), 72% cognitive impairment (MMSE <28 in 60%, clock-drawing test ≥3 in 63%) and 14% signs of depression in the GDS. A high number of patients (37.0%) revealed abnormal results in 5 of 6 CGA tests, whereas only 3.7%, 8.0%, and 6.3% respectively, had 0, or 1 or 2 abnormal tests. 22.3% and 15.0% respectively had 3 or 4 abnormal tests while a lower number (7.7%) exhibited 6 abnormal test results.

### Association with length of hospital stay

**Fig 2:**
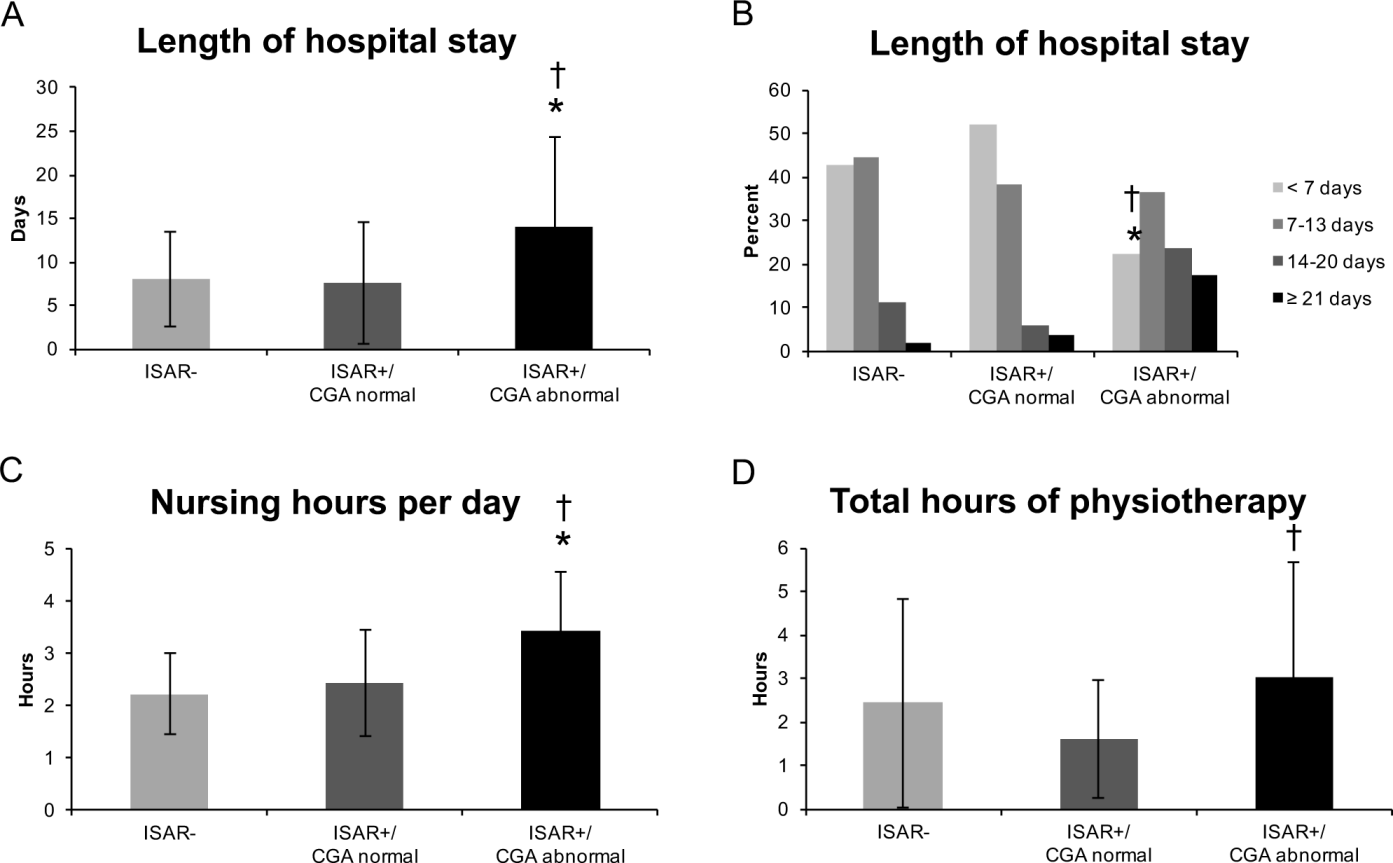
**Association of ISAR and CGA results with (A) Length of hospital stay in days, (B) Length of hospital stay in categories, (C) Nursing hours per day and (D) total hours of physiotherapy.** *p<0.001 compared with ISAR-, †p<0.001 compared with ISAR+/CGA normal. Abbreviations as in Table 1.

ISAR+/CGA abnormal patients stayed significantly longer in hospital than ISAR+/CGA normal and ISAR- patients (both p<0.001), while ISAR+/CGA normal and ISAR- patients did not significantly differ from each other (p = 0.912, Fig 2A). Since only patients with a hospital stay longer than a week typically qualify for geriatric rehabilitation, we also looked at different categories of durations of the hospital stay. This analysis revealed that a significantly smaller percentage of patients stayed shorter than 7 days in the ISAR+/CGA abnormal compared with ISAR+/CGA normal and ISAR- group (both p<0.001), confirming that abnormal CGA indicates geriatric rehabilitation needs (Fig 2B).

### Association with nursing hours

Patients with ISAR+/CGA abnormal received significantly more nursing hours per day than ISAR+/CGA normal and ISAR- patients (both p<0.001) (Fig 2C). The latter two groups did not significantly differ from each other (p = 0.426). For the total hours of nursing, which also depends on the length of the hospital stay, this difference was even more pronounced: ISAR+/CGA abnormal patients had more than twice the amount of total nursing hours (49.6±43.0) than ISAR+/CGA normal (19.1±18.0) and ISAR- (19.2±14.9) patients (both p<0.001).

### Association with hours of physiotherapy

ISAR+/CGA abnormal patients received significantly more total hours of physiotherapy during their hospital stay than ISAR+/CGA normal patients (p<0.001) (Fig 2D). All other groups did not differ from each other (p = 0.368 for ISAR+/CGA abnormal vs ISAR-, p = 0.189 for ISAR+/CGA normal vs ISAR-).

### Association with falls

Nineteen patients (5.0%) experienced a fall during their hospital stay. Of those, 18 (7.3%) were ISAR+/CGA abnormal, none (0%) ISAR+/CGA normal and 1 (1.9%) ISAR-. Almost twice as many falls occurred during nighttime (n = 12; 8 pm to 8 am) in comparison to daytime (n = 7). Sixteen falls took place in patients’ room, 2 in the bathroom and 1 on the corridor. Nine falls occurred while the patient was getting up from bed, 5 while walking, 4 while sitting down, and 1 during toileting. When patients were asked about their intention when the fall occurred, the majority (n = 8) reported that they were about to go to the toilet alone. Most of the falls did not lead to any medical consequences (n = 15), 2 had slight injuries (bruises and abrasion) and only 2 led to severe complications (1 proximal femur fracture and 1 humerus reosteosynthesis).

### Association with type of discharge

**Fig 3:**
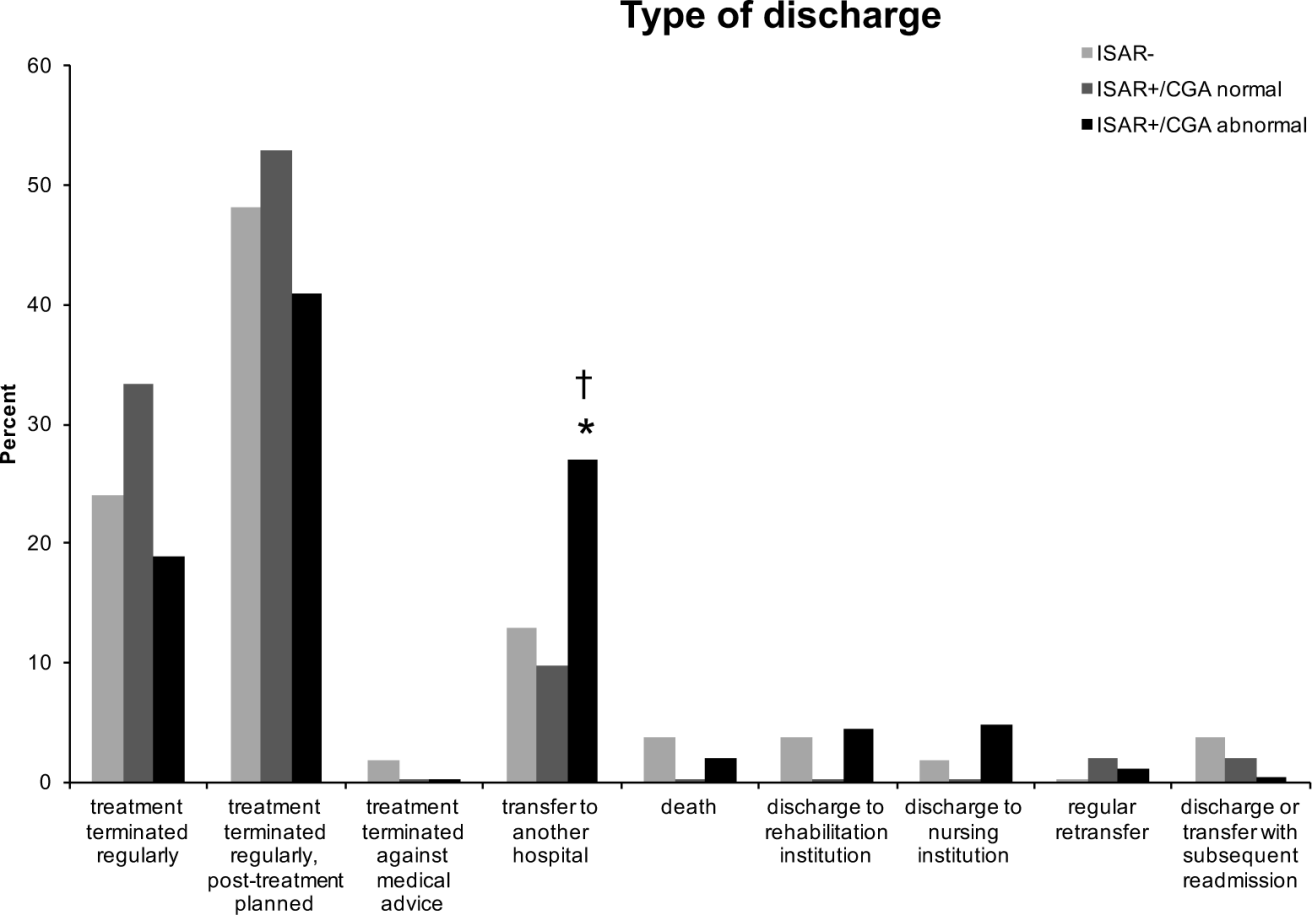
Association of ISAR and CGA results with type of discharge. *p<0.05 compared with ISAR-, †p<0.05 compared with ISAR+/CGA normal. Abbreviations as in Table 1.

The majority of patients finished their treatment regularly (64.5% of the total cohort) either with or without the necessity of later in-house post-treatment (e.g., implant removal or radiological control) (Fig 3). ISAR+/CGA abnormal patients (27.0%) had to be transferred to another hospital significantly more often than ISAR+/CGA normal (9.8%, p = 0.011) and ISAR- (13.0%, p = 0.036) patients. Death or discharge to a rehabilitation or nursing institution was rare (1.7%, 3.7% and 4.1%, respectively for the total cohort). There were no significant differences between groups.

### Predictors of length of hospital stay

In unadjusted regressions, younger age (β = -0.21, 95% CI = -0.32 to -0.10, p<0.001), male sex (B = 5.17, 95% CI = 2.51 to 7.82, p<0.001), high number of admission diagnoses (β = 0.38, 95% CI = 0.27 to 0.49, p<0.001), activities of daily living impairment (B = 7.01, 95% CI = 3.93 to 10.08, p<0.001), mobility impairment (B = 5.35, 95% CI = 2.29 to 8.41, p = 0.001) and presence of signs of depression (B = 4.53, 95% CI = 1.30 to 7.76, p = 0.006) predicted longer hospital stay, while all other variables did not have a significant effect (Table 3). In a multivariable regression including the factors age, sex and ISAR score (model 1), younger age and male sex predicted longer length of hospital stay. When age and sex were combined with mobility and cognition impairment and signs of depression in the CGA (model 2), all factors except for cognition impairment were significant predictors. Replacing mobility with activities of daily living impairment, which strongly depends on mobility, led to similar results (model 3). When both mobility and activities of daily living impairment were included (model 4), mobility impairment lost significance. Adding ISAR score to this model did not alter results (model 5). In contrast to this, adding the number of admission diagnoses revealed higher number of admission diagnoses as independent predictor of longer length of hospital stay (β = 0.28, 95% CI = 0.18 to 0.39, p<0.001, model 6) and improved model performance from R^2^ = 0.172 to 0.243. In summary, these analyses show that age, sex, number of admission diagnoses and CGA results (namely activities of daily living impairment and signs of depression), are independent predictors of the length of hospital stay, while the ISAR score is not.

**Table 3:** Predictors of total length of hospital stay in days for patients with ISAR+ receiving CGA (n = 300).

	Unadjusted			Model 1 Corrected R^2^ = 0.077			Model 2 Corrected R^2^ = 0.143			Model 3 Corrected R^2^ = 0.174		
	β or B	95% CI	p-value	β or B	95% CI	p-value	β or B	95% CI	p-value	β or B	95% CI	p-value
Age	-0.21	-0.32 to -0.10	<0.001	-0.20	-0.31 to -0.09	0.001	-0.25	-0.36 to -0.14	<0.001	-0.25	-0.35 to -0.14	<0.001
Sex (male vs female)	5.17	2.51 to 7.82	<0.001	4.61	1.95 to 7.28	0.001	3.99	1.42 to 6.57	0.002	4.08	1.55 to 6.60	0.002
Number of admission diagnoses	0.38	0.27 to 0.49	<0.001									
ISAR score	0.02	-0.09 to 0.14	0.708	0.10	-0.02 to 0.21	0.094						
Activities of daily living impairment (yes vs no)	7.01	3.93 to 10.08	<0.001							8.27	5.33 to 11.21	<0.001
Mobility impairment (yes vs no)	5.35	2.29 to 8.41	0.001				6.61	3.62 to 9.60	<0.001			
Cognition impairment (yes vs no)	0.28	-2.27 to 2.83	0.827				0.33	-2.09 to 2.76	0.788	0.33	-2.01 to 2.71	0.784
Signs of depression (yes vs no)	4.53	1.30 to 7.76	0.006				3.94	0.86 to 7.03	0.012	4.49	1.47 to 7.51	0.004
	Model 4 Corrected R^2^ = 0.174			Model 5 Corrected R^2^ = 0.172			Model 6 Corrected R^2^ = 0.243					
	β or B	95% CI	p-value	β or B	95% CI	p-value	β or B	95% CI	p-value			
Age	-0.25	-0.36 to -0.14	<0.001	-0.26	-0.37 to -0.15	<0.001	-0.24	-0.35 to -0.14	<0.001			
Sex (male vs female)	4.05	1.52 to 6.58	0.002	4.13	1.58 to 6.67	0.002	2.56	0.54 to 5.06	0.045			
Number of admission diagnoses							0.28	0.18 to 0.39	<0.001			
ISAR score				0.03	-0.08 to 0.15	0.550	0.02	-0.09 to 0.13	0.698			
Activities of daily living impairment (yes vs no)	7.05	3.00 to 11.10	0.001	7.01	2.95 to 11.06	0.001	5.72	1.82 to 9.63	0.004			
Mobility impairment (yes vs no)	1.77	-2.27 to 5.81	0.389	1.68	-2.38 to 5.74	0.417	1.18	-2.71 to 5.06	0.552			
Cognition impairment (yes vs no)	0.30	-2.01 to 2.68	0.806	0.20	-2.21 to 2.60	0.873	-0.20	-2.51 to 2.10	0.862			
Signs of depression (yes vs no)	4.35	1.31 to 7.39	0.005	4.25	1.19 to 7.31	0.007	3.72	0.79 to 6.65	0.013			

β, standardized regression coefficient; B, unstandardized regression coefficient; CI, confidence interval; other abbreviations as in Table 1.

### Predictors of nursing hours

In unadjusted regressions, high age (β = 0.14, 95% CI = 0.02 to 0.25, p = 0.018), high number of admission diagnoses (β = 0.34, 95% CI = 0.23 to 0.44, p<0.001), high ISAR score (β = 0.27, 95% CI = 0.16 to 0.38, p<0.001), activities of daily living impairment (B = 1.01, 95% CI = 0.66 to 1.36, p<0.001), mobility impairment (B = 0.85, 95% CI = 0.48 to 1.18, p<0.001) and cognition impairment (B = 0.52, 95% CI = 0.23 to 0.81, p<0.001) predicted higher number of nursing hours, while all other variables did not have a significant effect (Table 4). In a multivariable regression including the factors age, sex and ISAR score (model 1), only ISAR score predicted the number of nursing hours. When age and sex were combined with mobility and cognition impairment and signs of depression (model 2), mobility and cognition impairment were significant predictors. Replacing mobility with activities of daily living impairment again led to similar results (model 3). When both mobility and activities of daily living impairment were included (model 4), mobility impairment again lost significance. Adding ISAR score (β = 0.20, 95% CI = 0.09 to 0.32, p<0.001, model 5) and the number of admission diagnoses (β = 0.29, 95% CI = 0.18 to 0.39, p<0.001, model 6) to this model, improved model performance (from R^2^ = 0.116 to 0.150 to 0.222) and revealed that high number of admission diagnoses, high ISAR score and abnormal CGA results (namely activities of daily living and cognitive impairment) are independent predictors of increased nursing hours.

**Table 4:** Predictors of nursing hours per day for patients with ISAR+ receiving CGA (n = 300).

	Unadjusted			Model 1 Corrected R^2^ = 0.071			Model 2 Corrected R^2^ = 0.087			Model 3 Corrected R^2^ = 0.117		
	β or B	95% CI	p-value	β or B	95% CI	p-value	β or B	95% CI	p-value	β or B	95% CI	p-value
Age	0.14	0.02 to 0.25	0.018	0.08	-0.04 to 0.19	0.183	0.06	-0.06 to 0.17	0.322	0.06	-0.05 to 0.17	0.286
Sex (male vs female)	-0.06	-0.38 to 0.26	0.714	0.07	-0.24 to 0.39	0.647	0.01	-0.01 to 0.04	0.968	0.02	-0.29 to 0.32	0.920
Number of admission diagnoses	0.34	0.23 to 0.44	<0.001									
ISAR score	0.27	0.16 to 0.38	<0.001	0.26	0.14 to 0.37	<0.001						
Activities of daily living impairment (yes vs no)	1.01	0.66 to 1.36	<0.001							0.94	0.59 to 1.30	<0.001
Mobility impairment (yes vs no)	0.85	0.48 to 1.18	<0.001				0.75	0.39 to 1.11	<0.001			
Cognition impairment (yes vs no)	0.52	0.23 to 0.81	0.001				0.45	0.15 to 0.74	0.003	0.45	0.16 to 0.74	0.002
Signs of depression (yes vs no)	0.12	-0.26 to 0.51	0.523				-0.06	-0.43 to 0.32	0.768	0.01	-0.36 to 0.37	0.974
	Model 4 Corrected R^2^ = 0.116			Model 5 Corrected R^2^ = 0.150			Model 6 Corrected R^2^ = 0.222					
	β or B	95% CI	p-value	β or B	95% CI	p-value	β or B	95% CI	p-value			
Age	0.05	-0.06 to 0.17	0.346	0.02	-0.09 to 0.13	0.745	0.04	-0.07 to 0.15	0.508			
Sex (male vs female)	0.01	-0.29 to 0.32	0.935	0.07	-0.24 to 0.37	0.666	-0.12	-0.42 to 0.18	0.431			
Number of admission diagnoses							0.29	0.18 to 0.39	<0.001			
ISAR score				0.20	0.09 to 0.32	<0.001	0.19	0.08 to 0.30	0.001			
Activities of daily living impairment (yes vs no)	0.81	0.32 to 1.30	0.001	0.78	0.30 to 1.26	0.002	0.63	0.16 to 1.09	0.008			
Mobility impairment (yes vs no)	0.19	-0.30 to 0.68	0.436	0.13	-0.35 to 0.61	0.600	-0.07	-0.39 to 0.53	0.770			
Cognition impairment (yes vs no)	0.44	0.16 to 0.73	0.003	0.37	0.09 to 0.66	0.011	0.33	0.05 to 0.60	0.020			
Signs of depression (yes vs no)	-0.01	-0.38 to 0.36	0.958	-0.08	-0.45 to 0.28	0.657	-0.14	-0.49 to 0.20	0.414			

β, standardized regression coefficient; B, unstandardized regression coefficient; CI, confidence interval; other abbreviations as in Table 1.

### Predictors of hours of physiotherapy

In unadjusted regressions, younger age (β = -0.13, 95% CI = -0.26 to -0.01, p = 0.035), high number of admission diagnoses (β = 0.20, 95% CI = 0.08 to 0.32, p = 0.001), activities of daily living impairment (B = 1.54, 95% CI = 0.56 to 2.52, p = 0.002) and mobility impairment (B = 1.36, 95% CI = 0.43 to 2.28, p = 0.004) predicted higher number of physiotherapy hours, while all other variables did not have a significant effect (Table 5). When combined with sex and ISAR score in multivariable regressions (model 1), age revealed a tendency towards statistical significance. In the model including age, sex, mobility and cognition impairment and signs of depression (model 2), age and mobility impairment were significant predictors. Replacing mobility with activities of daily living impairment did reveal age and activities of daily living impairment as significant predictors (model 3). When both mobility and activities of daily living impairment were included (model 4), both variables lost statistical significance. Adding ISAR score to this model did not alter results to major extent (model 5), while further adding the number of admission diagnoses (β = 0.16, 95% CI = 0.04 to 0.29, p = 0.012, model 6) enhanced model performance (from R^2^ = 0.056 to 0.076). This demonstrates that younger age and high number of admission diagnoses, but not ISAR score or CGA results are predictors of total physiotherapy hours.

**Table 5:** Predictors of total hours of physiotherapy for patients with ISAR+ receiving CGA (n = 300).

	Unadjusted			Model 1 Corrected R^2^ = 0.008			Model 2 Corrected R^2^ = 0.047			Model 3 Corrected R^2^ = 0.050		
	β or B	95% CI	p-value	β or B	95% CI	p-value	β or B	95% CI	p-value	β or B	95% CI	p-value
Age	-0.13	-0.26 to -0.01	0.035	-0.12	-0.25 to 0.01	0.080	-0.18	-0.30 to -0.05	0.007	-0.17	-0.30 to -0.04	0.009
Sex (male vs female)	0.25	-0.50 to 1.01	0.512	0.08	-0.69 to 0.85	0.840	0.02	-0.74 to 0.78	0.958	0.06	-0.70 to 0.82	0.877
Number of admission diagnoses	0.20	0.08 to 0.32	0.001									
ISAR score	-0.08	-0.20 to 0.04	0.185	-0.05	-0.18 to 0.08	0.437						
Activities of daily living impairment (yes vs no)	1.54	0.56 to 2.52	0.002							1.79	0.80 to 2.78	<0.001
Mobility impairment (yes vs no)	1.36	0.43 to 2.28	0.004				1.66	0.71 to 2.62	0.001			
Cognition impairment (yes vs no)	-0.12	-0.81 to 0.57	0.733				-0.21	-0.90 to 0.49	0.554	-0.18	-0.87 to 0.51	0.605
Signs of depression (yes vs no)	0.31	-0.59 to 1.21	0.493				0.21	-0.68 to 1.11	0.641	0.33	-0.56 to 1.22	0.465
	Model 4 Corrected R^2^ = 0.054			Model 5 Corrected R^2^ = 0.056			Model 6 Corrected R^2^ = 0.076					
	β or B	95% CI	p-value	β or B	95% CI	p-value	β or B	95% CI	p-value			
Age	-0.18	-0.31 to -0.05	0.006	-0.16	-0.29 to -0.03	0.014	-0.16	-0.28 to -0.03	0.018			
Sex (male vs female)	0.04	-0.71 to 0.79	0.917	0.01	-0.75 to 0.77	0.980	-0.24	-1.02 to 0.53	0.536			
Number of admission diagnoses							0.16	0.04 to 0.29	0.012			
ISAR score				-0.08	-0.20 to 0.05	0.241	-0.08	-0.21 to 0.04	0.196			
Activities of daily living impairment (yes vs no)	1.16	-0.16 to 2.47	0.084	1.19	-0.12 to 2.50	0.075	1.00	-0.31 to 2.30	0.134			
Mobility impairment (yes vs no)	0.93	-0.33 to 2.19	0.147	0.95	-0.31 to 2.21	0.138	0.88	-0.37 to 2.12	0.169			
Cognition impairment (yes vs no)	-0.22	-0.91 to 0.47	0.532	-0.17	-0.87 to 0.53	0.632	-0.23	-0.92 to 0.47	0.522			
Signs of depression (yes vs no)	0.26	-0.64 to 1.15	0.572	0.32	-0.58 to 1.22	0.490	0.21	-0.68 to 1.10	0.642			

β, standardized regression coefficient; B, unstandardized regression coefficient; CI, confidence interval; other abbreviations as in Table 1.

### Predictors of in-hospital falls

Most likely due to the low number of fall events observed in our patient cohort, none of the variables evaluated above predicted falls in univariable and multivariable analyses (not shown).

## Discussion

In the geriatric consultation service at the Department of Orthopedics and Trauma Surgery of the University Hospital Essen we evaluated associations of ISAR score and CGA results with a variety of characteristics related to the hospital stay, namely the length of hospital stay, nursing and physiotherapy hours, and falls. Abnormal CGA resulted in an increased length of the hospital stay, an increased number of nursing hours, an increased number of physiotherapy hours, and an increased number of falls. In multivariable regression analyses, the CGA domains activities of daily living impairment and signs of depression predicted longer length of hospital stay. A high ISAR score and the CGA domains activities of daily living and cognitive impairment predicted increased nursing hours.

Of 423 patients ≥75 years admitted during a 10-months period, 381 (90.1%) received ISAR screening by the nursing staff, underlining the utility of ISAR in in-patient hospital environments. Of all ISAR screenings completed, 85.8% were positive. The percentage of ISAR+ patients in our study is higher than previously reported in the literature, however so far studies were only done in general emergency departments. In 1,632 patients ≥75 years (mean age: 84.0±5.5 years, 61% female) from a geriatric emergency department of the National Institute of Health and Science on Aging Hospital in Ancona, Italy, 75% of ISAR screenings were positive [20]. In a small cohort of 258 slightly younger patients (mean age: 79 years) attending an emergency department of the Mount Sinai Hospital in Toronto, Canada, only 61.2% had positive screenings[21]. In a cohort of 198 patients of similar age (mean age: 78 years, 55% female) attending the emergency department at Amager Hospital, Denmark, 68% were ISAR+[22]. Only in 202 patients (mean age: 77±8 years, 55% male) attending the emergency department of two hospitals in Pittsburgh, Pennsylvania, 84% of ISAR screenings were positive[23], which is more similar to our results.

In 327 ISAR+ patients, CGAs could be performed in 300 patients (91.7%), of which 248 (82.7%) were abnormal indicating significant impairment in activities of daily living (85%) plus impairment in one of the domains mobility (84%), cognition (72%) or signs of depression (14%). These numbers are higher than reported in literature previously. The Pittsburgh study by Suffoletto et al. (2016) observed impaired mobility in 65% and severe cognitive impairment in 23%[23]. Reasons for this difference compared to our results could be that all subjects and not only ISAR+ were analyzed and that cognitive impairment was only coded in cases of severe impairment while we also included mild cognitive impairment. In the Danish cohort[22], 16% of those with ISAR+ results had cognitive deficits which however were only assessed via medical records review. This number is similar to our cohort, while 22% had emotional problems[22], which is more than in our cohort. In 1507 patients ≥70 years (mean age: 81 years, 64% women) admitted to geriatric units at Yale New Haven Hospital, Connecticut, USA, 52% had activities of daily living impairment and 45% had cognitive impairment[24]. In this study, patients again did not receive an ISAR screening before CGA and cognitive impairment only comprised more severe deficits. In a study conducted within four emergency departments in the greater Cleveland area in the USA taking part in the Systematic Intervention for a Geriatric Network of Evaluation and Treatment (SIGNET) which included administration of the triage risk screening tool followed by geriatric assessment in case of positive screening in 28,437 patients ≥65 years who returned home, 43% had impaired mobility, 17% cognitive impairment and the same number like in our cohort suffered from signs of depression (14%)[25]. In contrast to our study, patients had already returned home when the CGA was performed indicating that the patients had less severe illness. In addition, mobility was only assessed via patient interview and cognition focused on delirium and confusion while the MMSE used in our study focusses on a broader spectrum of cognitive functions. Signs of depression, which showed very similar prevalence as in our study, were assessed in a similar way compared to our study by choosing 11 items from the GDS.

We demonstrated for the first time that high ISAR score and abnormal CGA results are strongly associated with increased length of the hospitalization stay, nursing and physiotherapy hours, and falls. In multivariable regression analyses, activities of daily living impairment and signs of depression predicted longer hospital stay in addition to younger age, male sex and higher number of admission diagnoses, whereas ISAR score was not predictive. Our data are in contrast to the above mentioned smaller and younger Danish cohort[22], in which a significant correlation between ISAR score and days spent in hospital (r = 0.36, p<0.001) was found. Yet, their correlation analysis included the total cohort while we only selected ISAR+ patients. In their study, the mean length of the hospital stay (6 days) was also shorter than in our cohort. Within the whole cohort of 381 patients receiving an ISAR screening in our study, ISAR score is also moderately correlated with length of hospital stay (r = 0.11, p = 0.038). A 2004 review about the association between age and length of hospital stay in elderly patients admitted to the acute hospital setting[26] showed that age was not related to length of hospital stay in 7 of the 9 studies[27–33]. In the other two 2 studies, in contrast to our study, higher age predicted longer stay[34,35]. In contrast to our study, sex was not associated with length of hospital stay in 6 of 7 studies included in the review[27–29,31,32,34] and in one study including 404 elderly patients (58% females) admitted to a geriatric service in Birmingham, female sex predicted longer hospital stay[30]. Supporting our observation, male sex predicted longer stay in hospital in 1123 patients (mean age: 82±7 years, 56% women) admitted to geriatric and acute internal medicine care wards of 7 Italian hospitals[36]. Differences in health care systems may explain diverging findings. Notably, all except 2 studies[27,33] of the review[26] revealed a significant association of cognition and length of hospital stay, whereas no study noted an association between depression and length of hospital stay. An association of the ability to perform activities of daily living and length of hospital stay has repeatedly been reported [27,30–32,36].

To the best of our knowledge, no study analyzed links between ISAR score, CGA results, and nursing and physiotherapy hours during acute hospital stay. For the first time we showed that in multivariable regression analyses higher ISAR score and cognitive impairment were independently associated with higher nursing hours per day, even when adjusted for age, sex and the number of admission diagnoses. It is notable that ISAR score predicts nursing hours in multivariable analyses, because it reveals that the nursing stuff, who performed the ISAR screening, has a reliable estimate of functional dependence of their patients. Similar to our results, a multi-center cohort study including 271 patients (mean age 67.0 years, range 18–100; 53.9% female) on acute hospital units providing rehabilitation care, showed that activities of daily living impairment was associated with increased nursing hours when adjusted for age and sex, which, like in our study, had no significant effect[37]. In contrast to nursing hours, higher number of physiotherapy hours were not predicted by ISAR score or CGA results, when information about the age, sex and the number of admission diagnoses was included into multivariable analyses. Without adjustment for the number of admission diagnoses, mobility impairment and activities of daily living impairment were independent predictors for physiotherapy hours. So far there is no literature on the association between patient characteristics and hours of physiotherapy. However it has been suggested that physical therapy is more influenced by patient diagnosis, experience, institutional practice, and surgeon preferences than individual patient characteristics[38].

The idea that an abnormal CGA indicates hospital-associated risks was strengthened by our observation that ISAR+ patients with abnormal CGA exhibited a higher percentage of falls than ISAR+ patients with normal CGA or ISAR- patients. Due to the low statistical power related to the low number of falls, we did not find predictors of falls in regression analyses. Importantly, most falls occurred during the nighttime when patients wanted to go to the toilet. Elderly patients had specific problems of adapting to foreign environment, and they prefer not to ask for help in order to keep their privacy[39]. Larger studies showed that cognitive impairment, which was highly prevalent in our cohort, increases the risk of falling. In a systematic review and meta-analysis by Muir et al. (2012) including 26 studies, the risk of falling in community-dwelling elderly ranged from 19–50% within 1 year follow-up and cognitive impairment was significantly associated with any fall, serious injuries and distal radius fractures[40]. In a population-based cross-sectional study of 6928 subjects ≥55 years, falls during the previous year also increased with memory impairment, and with female sex, disability, gait or postural disturbances, depressive episodes and with the number of drugs per day[41].

In view of the strong association of geriatric screening and CGA with length of hospital stay, nursing and physiotherapy hours, and falls during the hospital stay, future studies should further delineate consequences of abnormal ISAR and CGA results for long-term patient outcomes after hospital discharge such as mortality, rehospitalization, nursing home institutionalization, use of home healthcare services and quality of life. A limitation of our study is the cross-sectional design so that implications of abnormal ISAR and CGA for long-term outcomes after hospital discharge could not be assessed. A cross-sectional analysis of in-hospital death, short-term rehospitalization and referral to residential care facilities was not possible due to the small number of death and rehospitalization events and lack of information on residential care facility referral. Meta-analyses have already demonstrated benefits of CGA in the clinical setting for these outcomes. In a meta-analysis including 28 controlled trials with 4959 subjects receiving CGA and 4912 controls, institutional CGA reduced mortality, increased living at home, and improved cognitive function at 6 months[42]. In a later meta-analysis by Ellis et al (2011)[2], which analyzed the effect of mobile teams vs designated wards compared with usual care in 22 randomized controlled trials including 10,315 participants, beneficial effects on living at home were only observed when CGA was performed on geriatric wards and not by mobile teams in other environments. Yet, with regards to the combined outcome of death or deterioration and for the outcome cognitive function, both types of CGA had a beneficial effect after 1-year follow-up.

In a university orthopedics and trauma surgery environment we identified a high number of elderly patients at risk of adverse outcomes by ISAR screening and CGA, which is in accordance with previous literature. These patients exhibited high prevalence of impairment in activities of daily living, disturbed mobility, cognition and mood. To meet the needs of geriatric patients, future studies should systematically assess benefits for long-term outcome and adopt treatment strategies to patient risks. Interdisciplinary team approaches should be fostered to optimize patient management in elderly patients.
